# Impact of Enzymatic and Microbial Bioprocessing on Antioxidant Properties of Hemp (*Cannabis sativa* L.)

**DOI:** 10.3390/antiox9121258

**Published:** 2020-12-10

**Authors:** Erica Pontonio, Michela Verni, Cinzia Dingeo, Elixabet Diaz-de-Cerio, Daniela Pinto, Carlo Giuseppe Rizzello

**Affiliations:** 1 Department of Soil, Plant and Food Science, University of Bari Aldo Moro, Giovanni Amendola 165/A, 70126 Bari, Italy; michela.verni@uniba.it (M.V.); cdingeo@gmail.com (C.D.); 2 Department of Nutrition and Food Science, University of Granada, 18071 Granada, Spain; ediazdecerio002@correo.ugr.es; 3 Giuliani S.p.A., Pelagio Palagi, 2, 20129 Milan, Italy; dpinto@giulianipharma.com; 4 Department of Environmental Biology, “Sapienza” University of Rome, Piazzale Aldo Moro 5, 00185 Rome, Italy; carlogiuseppe.rizzello@uniroma1.it

**Keywords:** antioxidant activity, hemp, lactic acid bacteria, bioprocessing, proteolysis

## Abstract

Although the hemp seed boasts high nutritional and functional potential, its use in food preparations is still underestimated due to scarce technological properties and the presence of several anti-nutritional factors. Here, an optimization of a biotechnological protocol aimed at improving the antioxidant properties and the protein digestibility of the whole hemp seed has been proposed. Processing based on the use of commercial food grade enzymes and ad hoc selected lactic acid bacteria was tested and the phenolic and protein profiles were investigated through an integrated approach including selective extraction, purification, and identification of the potentially active compounds. The influence of the bioprocessing on the antioxidant activity of the hemp was evaluated both in vitro and on human keratinocytes. The lactic acid bacteria fermentation was the best method to significantly improve the antioxidant potential of the hemp through intense proteolysis which led to both the release of bioactive peptides and the increase in the protein digestibility. Moreover, changes in the phenolic profile allowed a significant protective effect against oxidative stress measured on the human keratinocyte cell line.

## 1. Introduction

*Cannabis sativa* L., commonly known as hemp, is an herbaceous, anemophilous plant belonging to the *Cannabaceae* family. *C. sativa* L. has recently drawn great interest due to its nutritional and pharmaceutical value, although, in the past, it has mainly been cultivated as a fiber crop and the seeds used as animal feed [1,2]

According to the proximal composition, hempseed contains approximately 25–30% oil and protein, and 30–40% fiber; however, this proportion is subjected to large variations among different hemp *cultivars* [3,4,5]. Moreover, hempseed is also rich in phenolic compounds, tocopherols, and phytosterols, which are natural antioxidants and may play a role in reducing the risk of chronic diseases [6].

The actual use of hempseed is mainly related to the oil, containing a high (>90%) proportion of unsaturated fatty acid and a desirable balance of ω-6/ω-3 fatty acids [2], and seed cake, mainly composed of protein (globulin and albumin), having high biological and functional value [7].

Despite its nutritive potential, the application of whole hempseed appears to be limited due to antinutritional factors and fibers content which may negatively affect the protein’s solubility, extractability, and hence its nutritional value [8]. Moreover, several research efforts have been made to exploit the hempseed functional features [2], for example through enzymatic hydrolysis of native proteins, aiming to releasing peptides with biological activity [1].

The release of antioxidant peptides [9], as well as the degradation of antinutritional factors [10,11], have largely been reported as being part of the activity of specific microbial groups, with lactic acid bacteria being the main actors. Indeed, they have widely been used as cell factories to improve the nutritional and functional features of plant-derived food matrices [10,11,12].

In this framework, the present study aimed at assessing the most suitable biotechnological protocol to improve the nutritional and functional properties of whole hempseed associated to the protein and phenolic fractions by exploiting enzymatic and fermentation treatments.

Proteases may be responsible for the release from the matrix of protein derivatives (mainly peptides) and phenols (by acting on protein–phenolics interactions) with strong functional properties [1,2,13]. In addition, xylanases, through the hydrolysis of insoluble fiber (mainly hemicellulose), can contribute to the release of proteins and phenols which are physically entrapped in the fiber matrix [13,14]. Both enzymatic treatments can lead to an increase in the antioxidant activity and protein digestibility of hempseed.

Ad hoc selected lactic acid bacteria [12] might act as (i) activators of endogenous proteolytic and cell-wall-degrading enzymes responsible for primary proteolysis and fibers hydrolysis, respectively [15,16,17]; (ii) cell factories for the release of antioxidant peptides through the complex proteolytic system [9], (iii) improvers of phenolic profiles [18,19]; and (iv) responsible for the decrease in antinutritional factors [11,12].

Commercial food-grade protease and xylanase and selected lactic acid bacteria were used in this study for hempseed processing. The characterization of raw and bioprocessed hempseed included biochemical analysis and in vitro plus cell-culture-based investigations in order to assess the antioxidant activity. Identification of peptides and phenols potentially responsible for the biological activity has also been carried out.

## 2. Materials and Methods

### 2.1. Raw Material, Enzymes and Microorganisms

Samples of whole hempseed (*Cannabis sativa* var. Futura75) were purchased from Leblè (Altamura, Italy). Prior to use, hempseeds were surface sterilized by soaking using a 15% (vol/vol) H_2_O_2_ solution as reported by Pontonio et al. [20]. Sterile demineralized water was used to wash the seeds after shaking and drying in a laminar flow hood for 1 h [20]. Seeds sterility was assessed by blotting them tightly on 1/10-strength tryptic soy agar and incubating plates at 30 °C for 48 h [20]. No microbial growth was found in 10 g of the sample. Surface-sterilized seeds were milled under nitrogen stream, dark and refrigerated conditions (circa 2 °C) using a laboratory mill Ika-Werke M20 (GMBH, and Co. KG, Staufen, Germany). Whole hemp flour had the following proximal composition: moisture, 6.5%; protein, 26.5% of dry matter (d.m); fat, 37.96% of d.m.; total dietary fiber, 29.52% of d.m.; ash, 5.98% of d.m.

Two commercial enzymatic food grade preparations, corresponding to the Depol761P (Biocatalysts Ltd., Wales, UK) (880 xylanase u/g) and VeronPS (protease activity 227 UHb/g) (AB Enzymes GmbH, Darmstadt, Germany), were used in this study. Selected lactic acid bacteria (LAB) belonging to the Culture Collection of the Department of Soil, Plant and Food Science, University of Bari Aldo Moro (Bari, Italy), previously isolated from hemp fermented flour and identified by partial sequencing of the 16S rRNA, recA and pheS genes as *Lactiplantibacillus plantarum* 18S9 and *Leuconostoc mesenteroides* 12MM1, were used for fermentation. The LAB strains were routinely cultivated on modified De Man, Rogosa and Sharpe (Oxoid, Basingstoke, Hampshire, UK) (mMRS, maltose and fresh yeast extract were added to MRS at 1 and 5%, respectively, and the final pH was 5.6) agar until the late exponential phase of growth was reached (circa 8 h), as previously determined by the analysis of the kinetics of growth [12]. Prior to fermentation, cells were harvested by centrifugation (10,000× *g*, 10 min, 4 °C), washed twice using 50 mM phosphate buffer, pH 7.0, and re-suspended in tap water.

### 2.2. Bioprocessing

A mixture of whole hemp flour and water (ratio 1:1, wt/wt) was either treated with enzymes or fermented by selected LAB. In detail, hemp flour and water were mixed in a 100 mL beaker, in an ice bath, and then the xylanase (H_X_) and protease (H_P_) were added at the doses of 1% (wt/wt of fiber) and 2.5% (wt/wt of protein), respectively, according to the manufacturer’s instructions. After manually mixing with a spatula, the headspace of the beaker was saturated with nitrogen in order to remove the dissolved oxygen and limit lipid oxidation. A similar approach was used for the fermented sample. *L. plantarum* 18S9 and *Leuc. mesenteroides* 12MM1 were both inoculated at a final cell density of circa 7.0 log10 cfu/g of sample (H_F_). All the samples were incubated for 24 h at 30 °C. A dough prepared as described above, without the addition of either lactic acid bacteria or enzymes, was used as control (H). Bioprocessing was carried out in triplicate.

### 2.3. Microbiological Analysis

For microbiological analysis, 10 g of dough were homogenized with 90 mL of sterile peptone water (1% [wt/vol] of peptone and 0.9% [wt/vol] of NaCl) solution. Presumptive LAB were enumerated using MRS (Oxoid) agar medium supplemented with cycloheximide (0.1 g/L). Plates were incubated under anaerobiosis (AnaeroGen and AnaeroJar, Oxoid) at 30 °C for 48 h. Cell densities of yeasts and molds were estimated on yeast peptone dextrose agar medium (YPDA) (Sigma-Merck, Darmstadt, Germany) supplemented with chloramphenicol (0.1 g/L), through pour and spread plate enumeration, respectively, and incubated at 25 °C for 72 h. The yeast/mold identification was done by visual analysis of colony morphology. Plate count agar (PCA, Oxoid), incubated at 30 °C for 48 h, was used to enumerate the total mesophilic bacteria (TMB), while violet red bile glucose agar (VRBGA, Oxoid), incubated at 37 °C for 24 h, was used to determine the total *Enterobacteriaceae*.

### 2.4. Biochemical Characterization

#### 2.4.1. pH and Organic Acids

A pHmeter M.507 (Crimson, Milan, Italy) equipped with a food penetration probe was used to measure the values of pH of doughs. Total titratable acidity (TTA), expressed as the amount (mL) of 0.1 M NaOH to achieve the pH of 8.3, was measured on 10 g of samples homogenized with 90 mL of distilled water.

Water/salt-soluble extracts (WSEs) from doughs were prepared according to the method described by Weiss et al. [21]. Briefly, doughs containing 1 g of hemp flour were suspended in 4 mL of 50 mM Tris–HCl (pH 8.8), incubated at 4 °C for 1 h under stirring conditions (150 rpm) and centrifuged at 12,000× *g* for 20 min. The supernatant was used for the determination of organic acid concentration by high-performance liquid chromatography (HPLC), using an ÄKTA Purifier system (GE Healthcare, Buckinghmshire, UK) equipped with an Aminex HPX-87H column (ion exclusion, Biorad, Richmond, CA, USA), and an UV detector operating at 210 nm. Elution was at 60 °C, with a flow rate of 0.6 mL/min, using H_2_SO_4_ 10 mM as the mobile phase [22].

#### 2.4.2. Proteins and Protein Derivatives

WSEs, obtained as reported above, were used for the quali-quantitative analysis of proteins, peptides and total free amino acids (TFAAs).

The concentration of proteins was determined by the Bradford method [23]. Two-dimensional electrophoresis (2-DE) was carried out with the immobiline-polyacrylamide system, as described by Di Cagno et al. [24]. Aliquots of proteins (120 μg) were used for the electrophoretic run. Isoelectric focusing (IEF) was carried out on immobiline strips. The first dimension was carried out on immobilized-pH-gradient [IPG] strips (Amersham Pharmacia Biotech, Uppsala, Sweden), under a nonlinear pH gradient from 3.0 to 10.0, by using an IPG-phore (Amersham Pharmacia Biotech, Uppsala, Sweden) at 20 °C. The second dimension was carried out in a Laemmli system on 15% polyacrylamide gels (13 cm by 20 cm by 1.5 mm) at a constant current of 30 mA/gel and at 15 °C for approximately 5 h, until the dye front reached the bottom of the gel. 2-DE protein standards (Biorad, Richmond, CA, USA) were used for isoelectric point (pI) and molecular weight estimation. Gels were silver-stained [25]. Image analysis of the gels, acquired by a gel scanner (Amersham Pharmacia Biotech, Uppsala, Sweden), was carried out with the University of Texas Health Science Centre—San Antonio (UTHSCSA) ImageTool software (version 2.0; available from maxrad6.uthscsa.edu).

The analysis of peptides was made on WSEs and methanolic extracts (MEs). MEs were obtained by mixing 5 g of each dough with 50 mL of 80% methanol. The mixture was purged with nitrogen stream for 30 min, under a stirring condition, and centrifuged at 6490 rpm for 20 min. MEs were transferred into test tubes, purged with nitrogen stream and stored at circa 4 °C before analysis.

Aiming at removing proteins and FAA, WSEs and MEs were treated with trifluoroacetic acid (0.05% wt/vol) and subjected to dialysis (cut-off 500 Da), respectively. Then, peptide concentration was determined by the *o*-phtaldialdehyde (OPA) method as described by Church et al. [26]. Peptides profiles in WSE were determined by reversed-phase fast performance liquid chromatography (RP-FPLC), using a Resource RPC column and ÄKTA FPLC equipment, with the UV detector operating at 214 nm (GE Healthcare Bio-Sciences AB, Uppsala, Sweden). In particular, sample aliquots containing 1 mg/mL of peptides were loaded onto the column. Gradient elution was performed at a flow rate of 1 mL/min using a mobile phase composed of water and acetonitrile (CH_3_CN), containing 0.05% TFA. The concentration of CH_3_CN was increased linearly from 5 to 46% between 16 and 62 min, and from 46 to 100% between 62 and 72 min. TFAAs were analyzed by a Biochrom 30 series Amino Acid Analyzer (Biochrom Ltd., Cambridge Science Park, UK) with a Na-cation-exchange column (20 by 0.46 cm internal diameter), as described by Rizzello et al. [22].

#### 2.4.3. Protein Digestibility

The in vitro protein digestibility (IVPD) was determined by the method proposed by Akeson and Stahmann [27] with some modifications [28] by mimicking in vivo digestion in the gastro-intestinal tract. IVPD was expressed as the percentage of the total protein which was solubilized after enzyme hydrolysis. The concentration of protein was determined by the Bradford method as reported above.

### 2.5. In Vitro Antioxidant Activity

The radical DPPH was used for determining the free radical scavenging activity (RSA) [22] on the WSEs and MEs. According to the method reported by Yu et al. [29], the RSA was determined using the stable 2,2-diphenyl-1-picrylhydrazyl radical (DPPH). The RSA was expressed as follows: DPPH scavenging activity (%) = [(blank absorbance—sample absorbance)/blank absorbance] × 100. The value of absorbance was compared with 75 ppm butylated hydroxytoluene (BHT), which was used as the antioxidant reference.

### 2.6. Characterization of the Antioxidant Compounds

#### 2.6.1. Purification of Antioxidant Peptides

WSEs obtained from H_F_, which showed the highest RSA, were automatically fractionated (2 mL per fraction, 33 fractions for each run) by reversed-phase fast performance liquid chromatography (RP-FPLC), under the conditions described above for the peptide analysis. Fractions were freeze-dried to remove solvents and re-dissolved in sterile water to determine the peptide concentration through the OPA method. Each fraction was also subjected to in vitro assays for RSA using the radical DPPH, as reported above.

#### 2.6.2. Stability to Digestion and Thermal Treatments

WSEs and purified fractions, showing the strongest RSA, were subjected to sequential protein hydrolysis by digestive enzymes (pepsin, pancreatin, and trypsin) according to the method described by Pasini et al. [30]. After treatments, samples were subjected to the scavenging activity determination on radical DPPH, as described above.

#### 2.6.3. Identification of Antioxidant Peptides

The peptides contained in the WSE fractions with the highest radical-scavenging activity were further purified and identified. The analysis was carried out by nano-liquid chromatography-electrospray ionisation-mass spectra/mass spectra (nano-LC-ESI-MS/MS), using a Finnigan LCQ Deca XP Max ion trap mass spectrometer (Life Technologies GmbH, Darmstadt, Germany) through the nano-ESI interface. According to manufacturer’s instrument settings for nano-LC-ESI-MSMS analyses, MS spectra were automatically taken by Xcalibur software (Life Technologies GmbH, Darmstadt, Germany), in positive ion mode. MS/MS spectra were processed using the software BioWorks 3.2 (Life Technologies GmbH, Darmstadt, Germany), generating peak lists suitable for database searches. Peptides were identified using MS/MS ion search of the Mascot search engine (Matrix Science, London, England) and NCBIProtdatabase (National Centre for Biotechnology Information, Bethesda, MD, USA). For the identification of peptides, the following parameters were considered: enzyme: “none”; instrument type: “ESI-trap”; peptide mass tolerance: ±0.1% and fragment mass tolerance: ±0.5 Da. Results from peptide identification were subjected to a manual evaluation, as described by Chen et al. [31], and the validated peptide sequences explained all the major peaks in the MS/MS spectrum.

#### 2.6.4. Extraction of Phenolic Compounds and Qualitative and Quantitative Analysis by UPLC-PDA-ESI-QTOF

Free and bound phenolics were extracted as described in Verardo et al. [32]. Briefly, 4 g of freeze-dried raw and bioprocessed hemp were extracted twice in an ultrasonic bath with ethanol/water (4:1 vol/vol) for 10 min. The supernatants were collected, evaporated at 40 °C in a rotary evaporator, and reconstituted with 2 mL of methanol/water (1:1 vol/vol). The extracts were stored at −18 °C until use. Residues of free phenolics extraction were digested with 300 mL of 2 M NaOH at room temperature overnight by shaking under nitrogen gas. At the end of the incubation, the mixtures were acidified (pH 2–3) with hydrochloric acid and extracted diethyl ether/ethyl acetate (1:1 vol/vol). The organic fractions were pooled and evaporated to dryness at 40 °C in a rotary evaporator, and bound phenolic compounds were reconstituted in 2 mL of methanol/water (1:1 vol/vol) as well. The identification and quantification of raw and bioprocessed hemp free and bound polyphenols was carried out with the use of an ACQUITY Ultra Performance LC system equipped with photodiode array detector with a binary solvent manager (Waters Corporation, Milford, MA, USA) series with a mass detector Q/TOF micro mass spectrometer (Waters) equipped with an electrospray ionization (ESI) source operating in negative mode with spectra acquired over a mass range from m/z 50 to 1100. The HPLC column was a fused-core Poroshell 120, SB-C18 (3.0 × 100 mm, 2.7 μm) from Agilent Technologies (Agilent Technologies, Palo Alto, CA, USA). The mobile phase and gradient program were used as previously described by Gómez-Caravaca et al. [33]. All solvents were filtered with a 0.45 mm filter disk. The compounds were monitored at 280 nm. MassLynx 4.1 software (Waters Corporation, Milford, MA, USA) was used to integrate and elaborate data. Aiming at quantifying the phenolic compounds, solutions of ferulic acid, chlorogenic acid, catechin and quercetin in methanol were prepared and used as standard.

### 2.7. Antioxidant Activity on Human Keratinocytes Cell Cultures

#### 2.7.1. Cytotoxicity

Human keratinocytes (NCTC2544) were cultured under humidified atmosphere (5% CO_2_, 37 °C), using Mammalian Cell Culture Media (RPMI Media 1640, Merck Life Science S.r.l., Milan, Italy), which was supplemented with 10% (wt/vol) fetal bovine serum (FBS), 2 mM glutamine, 1% penicillin (10,000 U/mL)/streptomycin (10,000 U/mL) mixture, and 0.1% gentamycin. The culture medium was renewed every two days. The cultures were used for viability assays after four passages.

Cell viability was measured using the MTT (3-(4,5-dimethyl-2-yl)-2,5-diphenyltetrazolium bromide) method [34]. The capacity of succinate dehydrogenase to convert 3-(4,5-dimethylthiazol-2-yl)-2,5-diphenyltetrazolium bromide into visible formazan crystals was assessed. For MTT assay, cells were seeded into a 96-well plate (Becton Dickinson France S.A., Meylan Cedex, France) at the density of 5 × 10^4^ cells/well and incubated for 24 h, when approximately 80% confluence was reached.

To determine the non-cytotoxic concentration, cells were treated with either WSE and ME of H and H_F_ and incubated for 24 h. Solvents in the extracts were previously removed through freeze-drying. The concentrations of freeze-dried WSE- and ME- H and H_F_ in the reaction mixture were 0.1, 1.0, and 10 mg/mL. Basal medium, without addition of either WSE- or ME- H and H_F_, was used as control. After incubation, medium was removed from each well and 100 µL of MTT (0.5 mg/mL final concentration) incubated (37 °C, 5% CO_2_) in the dark for 3 h. Finally, 100 µL of dimethyl sulphoxide (DMSO) were added to dissolve crystals. The solution was shaken at 120 rpm in the dark for 15 min at room temperature. The absorbance of the solution was read at 570 nm by a microplate reader (BioTek Instruments Inc., Bad Friedrichshall, Germany).

#### 2.7.2. Protective Effect on Oxidative-Induced Stress

The viability of H_2_O_2_-stressed human keratinocytes cells (NCTC2544) was determined by using MTT assay as described by Coda et al. [35] with some modifications. In this case, cells were incubated with either WSE- and ME- H and H_F_ for 16 h. According to the non-cytotoxic concentration, freeze-dried WSE- and ME- H and H_F_ in the reaction mixture were dissolved at 0.01 and 0.1 mg/mL. RPMI medium with 2.5% FBS, 2 mM glutamine, 1% penicillin (10,000 U/mL), streptomycin (10,000 U/mL) and 0.1% gentamycin was used as substrate. α-tocopherol (100, 250 and 500 µg/mL) was used as the positive control. The medium was removed from each well after the treatment and cells were exposed to 1 mM H_2_O_2_ for 90 min after washing. Hydrogen peroxide solution was prepared in RPMI medium without FBS. Two controls, one without the addition of WSE- and ME- H and H_F_, and another without H_2_O_2_ treatment, were included in the analysis. After the incubation, the MTT assay was performed as reported above. Data were expressed as the mean percentage of viable cells compared to the control culture, not exposed to oxidative stress. Each experiment was carried out in triplicate.

### 2.8. Statistical Analysis

All the chemical and physical analyses were carried out in triplicate for each batch of samples. Data were subjected to one-way ANOVA; pair-comparison of treatment means was achieved by Tukey’s procedure at *p* < 0.05, using the statistical software Statistica 12.5 (StatSoft Inc., Tulsa, OK, USA).

## 3. Results

### 3.1. Microbiological Analysis

Table 1 summarizes the microbiological and biochemical characterization of raw and bioprocessed hemp doughs. The cell density of TMB was the lowest in the raw hemp dough (H) being circa 3 log10 cfu/g. When the enzymes were used (H_P_ and H_X_), the incubation at 30 °C for 24 h caused an increase in the TMB of circa 2 log10 cfu/g. The highest value was found in fermented hemp (H_F_), due to the inoculum of the selected starters. The incubation led also to an increase in presumptive LAB and *Enterobacteriaceae* (up to circa 1 log10 ufc/g) in samples supplemented with xylanase and protease (Table 1). When selected LAB were used as starters, the final cell density in H_F_ reached circa 9 log10 cfu/g with an increase of circa 2 log10 cfu/g as compared to the initial inoculum, while *Enterobacteriaceae* remained almost stable, at circa 4.5 log10 ufc/g (Table 1). Yeasts and molds increased up to circa 1 log10 ufc/g in all samples compared to H, regardless of the bioprocess.

### 3.2. Bioprocessing

#### 3.2.1. Acidification

Statistically significant increases in organic acids concentration, both lactic and acetic, were found in all samples after incubation, as compared to H and regardless of the bioprocess used. As expected, according to the LAB cell density, the highest values and increases were found for H_F_ (Table 1). Decreases in pH values in H, H_P_ and H_X_ were found, probably due to the microbial contaminants of the samples. However, the highest decrease was found for the H_F_ (Table 1).

#### 3.2.2. Proteins and Protein Derivatives

**Figure 1:**
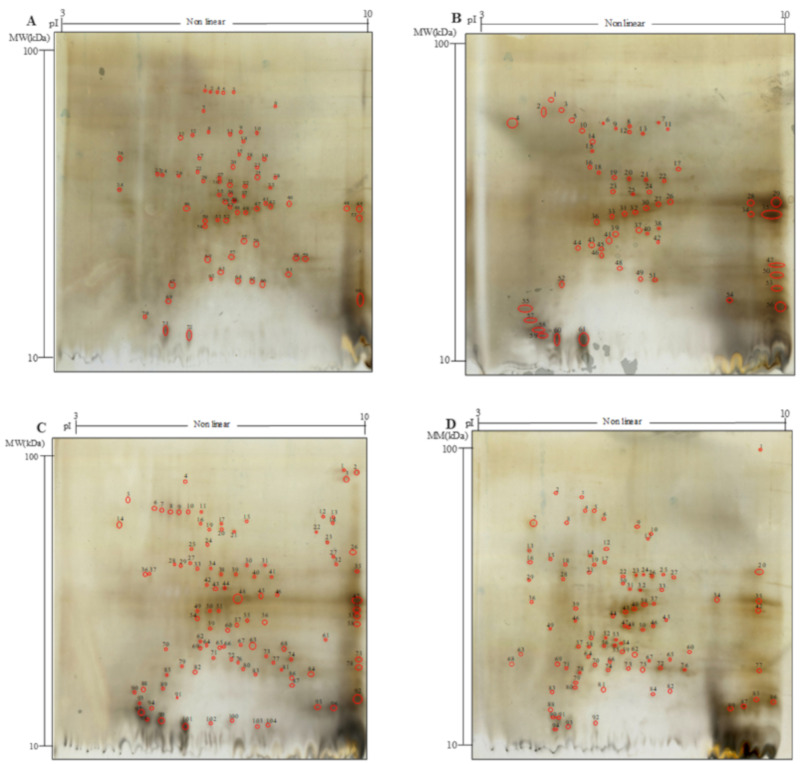
Two-dimensional gel electrophoresis (2-DE) analysis of the water/salt soluble proteins of raw and bioprocessed hemp. Panel (**A**), untreated hemp dough (H); (**B**), hemp dough treated with xylanase Depol 761P (1% wt/wt of fiber) (H_X_); (**C**), hemp dough treated with protease VeronPS (2.5% wt/wt of protein) (H_P_); (**D**), hemp dough fermented by *Lactiplantibacillus plantarum* 18S9 and *Leuconostoc mesenteroides* 12MM1 (ratio 1:1, final cell density of circa 7 log10 cfu/g) (H_F_). All treated samples were incubated at 30 °C for 24 h.

**Figure 2:**
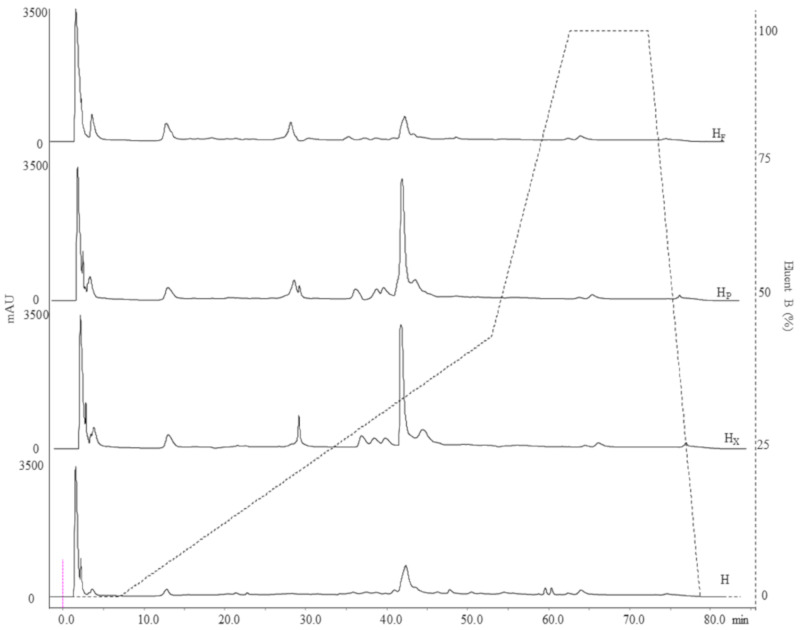
Peptide profiles of raw and bioprocessed hemp obtained by RP-FPLC (detector at 214 nm). Dashed line refers to the eluent B gradient. H, untreated hemp dough; H_X_, hemp dough treated with xylanase Depol 761P (1% wt/wt of fiber); H_P_, hemp dough treated with protease VeronPS (2.5% wt/wt of protein); H_F_, hemp dough fermented by *Lactiplantibacillus plantarum* 18S and *Leuconostoc mesenteroides* 12MM1 (ratio 1:1, final cell density of circa 7 log10 cfu/g). All treated samples were incubated at 30 °C for 24 h.

Aiming at investigating the effect of bioprocessing on H protein profile, two-dimensional electrophoresis (2-DE), and liquid chromatography were used to evaluate proteins and polypeptides with molecular weight (MW) ranging between 10 and 200 kDa, peptides with MW < 10 kDa and TFAA, respectively (Table 1, Figure 1A–D and Figure 2).

Overall, the enzymatic and microbial bioprocessing affected the protein profile and peptides and TFAA contents of hemp, with different magnitudes according to the bioprocess used.

Hempseed contains mainly storage proteins consisting of albumin (25–37%) and the legumin called edestin (67–75%) [36]; therefore, 2-DE analysis was performed only on the WSE (Figure 1A–D). Significant variations in the total number and distribution of polypeptides were observed (Figure 1 and Table S1). In detail, H was characterized by 68 spots mainly having MW between 20 and 37 kDa and 5.4 < pI < 7.3 (Figure 1A and Table S1). When xylanase was used (H_X_), the total spot number was lower than that of H and overall was the lowest among samples (67 spots), yet the distribution of the spots in the electrophoretic map changed. On the contrary, when protease (H_P_, 106) and selected lactic acid bacteria (H_F_, 98) were used, a higher number of spots as compared to H was found; indeed, 38 and 30 more spots were found, respectively (Figure 1A,B,D, Table S1). The bioprocess also influenced the distribution of the polypeptides according to the MW (Figure 1 and Table S1). The number of spots with MW in the range 20–37 kDa was lower in processed samples (30 and 35 in H_X_, and H_F_, respectively) as compared to H (40). Meanwhile, increases in polypeptides with MW below 20 kDa were found in H_X_, H_F_ and H_P_ (up to 62, 152 and 176%, respectively) as compared to H. According to the isoelectric focusing, more spots with pI 3–5.4 and 7.3–10 were observed in all bioprocessed samples compared to H (Table S1).

The 2-DE analysis was flanked by the study of peptide profiles (MW < 10 kDa). RP-FPLC chromatographic analysis (214 nm) highlighted significant differences in the peptide fraction among samples. Chromatograms revealed 63 (H), 56 (H_X_), and 46 (H_F_ and H_P_) peptide peaks (Figure 2). Although the total number of detected peaks decreased after bioprocessing, the total area of peaks was subjected to significant increases. Indeed, values of 3670, 4983, 7033 and 6625 mAU/mL were found for H, H_F_, H_P_ and H_X_, respectively (Figure 2). The highest value and increase were found when protease was used (circa 90%) followed by H_X_ (circa 80%) and H_F_ (circa 36%). When enzymes were used, changes were observed mainly in the range of 8 to 35% of the Eluent B gradient, while the hydrophilic (0–8% Eluent B) zone underwent minor alterations (Figure 2). Fermentation with selected LAB led to main changes in the range 0–20% of the Eluent B (Figure 2). In accordance with the increase in the total peak area, the concentration of peptides (measured by OPA) increased up to circa 40% (H_P_) when hemp flour was bioprocessed (Table 1).

An intense proteolysis often leads to the release of free amino acids, and hence the evaluation of the process as results of both the enzymatic and microbial treatments was integrated with the determination of the TFAA. The concentrations of TFAA in bioprocessed hemp ranged from 4843 ± 47 (H_X_) to 5198 ± 51 (H_F_) mg/kg and were significantly higher than that of H (1656 ± 23 mg/kg) (Table 1).

#### 3.2.3. In Vitro Protein Digestibility

**Figure 3:**
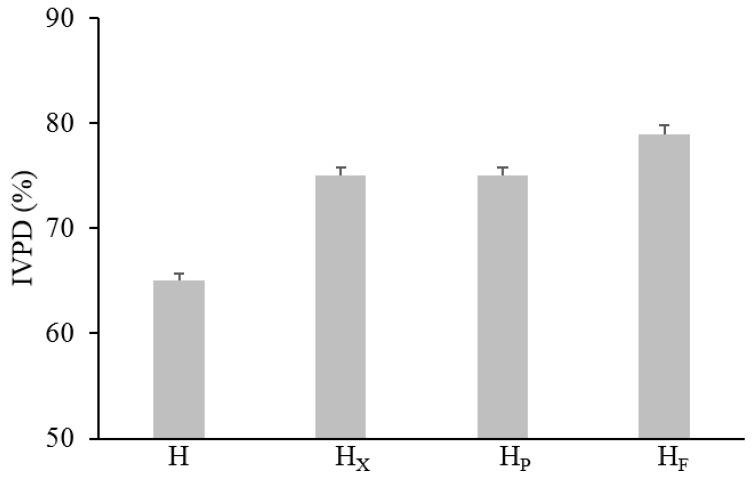
In vitro protein digestibility of raw and bioprocessed hemp. H, untreated hemp dough; H_X_, hemp dough treated with xylanase Depol 761P (1% wt/wt of fiber); H_P_, hemp dough treated with protease VeronPS (2.5% wt/wt of protein); H_F_, hemp dough fermented by *Lactiplantibacillus plantarum* 18S9 and *Leuconostoc mesenteroides* 12MM1 (ratio 1:1, final cell density of circa 7 log10 cfu/g). All treated samples were incubated at 30 °C for 24 h. All treated samples were incubated at 30 °C for 24 h.

The effect of bioprocessing on protein digestibility was investigated by mimicking the gastrointestinal digestion with an in vitro multi-step enzymatic treatment. Overall, it was found that hemp treatments led to IVPD increases proportional to the proteolysis degree (release of protein derivatives such as peptides and free amino acids). In detail, significant increases from circa 15 to 25% were found when enzymes and selected LAB were used, respectively (Figure 3). However, the highest IVPD value was found when LAB were used as starters (79%).

#### 3.2.4. In Vitro Radical Scavenging Activity

**Figure 4:**
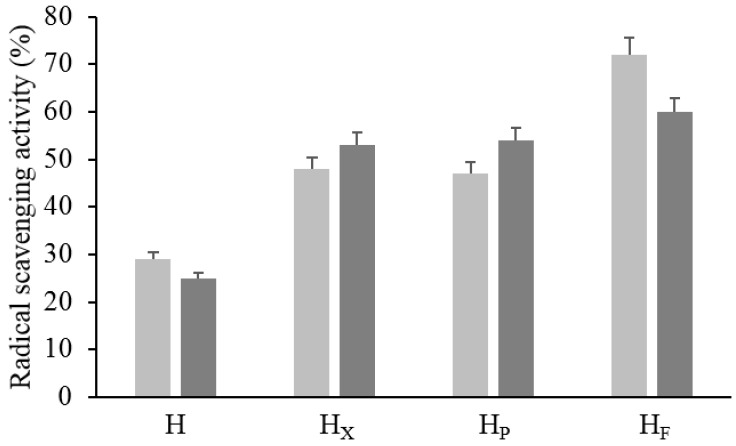
Radical scavenging activity (RSA) of raw and bioprocessed hemp. H, untreated hemp dough; H_X_, hemp dough treated with xylanase Depol 761P (1% wt/wt of fiber); H_P_, hemp dough treated with protease VeronPS (2.5% wt/wt of protein); H_F_, hemp dough fermented by *Lactiplantibacillus plantarum* 18S9 and *Leuconostoc mesenteroides* 12MM1 (ratio 1:1, final cell density of circa 7 log10 cfu/g). All treated samples were incubated at 30 °C for 24 h. RSA was determined in the methanolic (light grey) and water/salt (dark grey) extracts.

The most active antioxidant molecules in plant matrices mainly belong to the chemical classes of phenols and peptides and thus the ME and WSE, selectively accumulating the two groups of compounds, respectively, were subjected to the analysis of the RSA (Figure 4). Significantly higher activity was found in bioprocessed hemp as compared to H. Overall, the highest increases in the RSA were found in the WSEs (circa 112–140%), while the highest values (up to circa 70%) were found in the MEs. The use of selected LAB caused the highest increase in the values of the RSA in both WSE and ME (Figure 4).

### 3.3. Identification of the Antioxidant Compounds

#### 3.3.1. Peptides Identification

The RSA of the WSE of H_F_ was affected by enzymatic digestion and heating (up to circa 20%). Thirty-three fractions with a concentration in the range 0.37 ± 0.021–13.09 ± 0.04 mg/mL were obtained after WSE purification by RP-FPLC.

Only fractions (coded as 1, 2, 5 and 7) showing RSA higher than 50% were further characterized. The RSA ranged from 50.2 ± 1.1 (fraction n.7) to 67.1 ± 1.4% (fraction 2). Fractions were eluted at 0% (1 and 2), 6% (5) and 20% (7) of eluent B and were characterized by peptide concentration ranging from 4.99 ± 0.07 to 9.12 ± 0.03 mg/mL.

Five peptides, having 9–24 amino acid residues, were identified by nano-LC-ESI-MS/MS analysis. Single peptides were identified in fractions 1, 2 and 5, corresponding to ALASIGKATR (molecular weight of 987 Da), IGQSHPQALMYPLLVACKSISNLR (molecular weight of 2640 Da), and AQVSVGGGR (molecular weight of 829 Da), respectively (Table 2). According to the NCBI dataset, the peptides were encrypted into sequences of *Cannabis sativa* proteins nephrocystin-3, serine/threonine-protein kinase TOR and edestin 3, respectively. Two peptides were identified in the fraction 7 as having a sequence of AIENGAVSVSEPEEK (molecular weight of 1559 Da) and DLQIIAPSR (molecular weight of 1012 Da) and were encrypted into the sequences of an uncharacterized *C. sativa* protein and edestin3, respectively. All peptides identified were characterized by a content of hydrophobic amino acids higher than 50%, except for the peptide AIENGAVSVSEPEEK which showed a content of 44%. Moreover, all peptides contained branched-chai amino acid (Leucine, Isoleucine and Valine) with abundances ranging from 19 (AIENGAVSVSEPEEK) to 33% (DLQIIAPSR) (Table 2).

#### 3.3.2. Phenolic Profile

The free and bound polar extracts obtained from raw and bioprocessed hemp were analyzed using UPLC coupled to ESI-QTOF-MS in negative ionization mode. Table 3 summarizes the information related to the identification of the 81 compounds identified among free and bound profiles.

In the free phenolic profile, two isomers with *m/z* 315 were identified according to López-Cobo et al. [37] as protocatechuic acid glucosides. Isomers 5, 7, and 10–12 (*m/z* at 387) provided two fragments at *m/z* 163 and 207, which matched with the MS/MS fragments identified by Rodríguez-Pérez et al. [38] for tuberonic acid glucoside; however, this compound was not previously described in the *Cannabaceae* family.

Four phenolic acid derivatives, compounds 3 (*m/z* at 109), 4 (*m/z* at 135), 6 (*m/z* at 149) and 9 (*m/z* at 119), were respectively identified as catechol, 4-vinyl catechol, 4-vinyl guaiacol and 4-vinyl phenol, products of the decarboxylation of phenolic acids [39].

Two isomers with *m/z* 298 were identified as *N*-caffeoyltyramine, polyphenols previously found in hemp [40], whereas compound 14, at *m/z* 285 showing molecular formula C_15_H_9_O_6_, was identified as luteolin [41]. Seven isomers with *m/z* 373, showing molecular formula C_22_H_30_O_5_, were identified according to Frassinetti et al. [40] as cannabielsoic acid, as well as multiple isomers of cannabisin B (*m/z* at 595), C (*m/z* at 609), and D (*m/z* at 623).

Compounds 26–28 (*m/z* at 329), 40–41 (*m/z* at 277) and 42 (*m/z* at 279), were tentatively identified as fatty acids. Hempseeds consist of more than 30% oils, most of which are composed by polyunsaturated fatty acids, particularly linoleic (ω-6) and α-linolenic (ω-3) acids [38,40].

In the bound profile, two isomers showing *m/z* at 191 were identified as citric acid, while compounds 3 and 4, showing *m/z* at 331 and 315, were tentatively identified as protocatechuic acid derivative and glucoside, respectively (Table 3). The peak at *m/z* 289 was detected as catechin and its identity was corroborated by co-elution with a standard solution. Compound 8, with molecular ion [M-H]^−^ *m/z* 355, also providing fragments at *m/z* 337, 311 and 267, was identified as xerocomic acid [42]. Two isomers showing *m/z* at 163, providing a fragment at *m/z* 119 (C_8_H_8_O), corresponding to the loss of CO_2_, were identified as *trans* and *cis p*-coumaric acids [43], whereas two more compounds showing *m/z* at 327 and fragments in MS^2^ signal at *m/z* 163 and 119 were tentatively identified as coumaric acid derivatives. Compounds 13, 14 and 15, with *m/z* 369, providing fragments at *m/z* 377 and 265, were tentatively identified as flavodic acid isomers. Compound 20, showing *m/z* at 312 and providing fragments at *m/z* 190 (C_8_H_11_O) corresponding to the loss of ethyl phenol, 135 (C_8_H_7_O_2_), and 178 (C_9_H_8_NO_3_), was identified as *N*-*trans*-feryroyltyramine, previously found in hemp [44].

As for free compounds, *N*-caffeoyltyramine, providing fragments at *m/z* 178 and 135, and multiple cannabisin B, C and D isomers were also identified. Among hydrolysable phenolics, compounds 33 and 34, with molecular formula C_35_H_34_N_2_O_8_, were identified as isomers of cannabisin E, also providing fragments in the MS^2^ signal at 489 *m/z* (C_28_H_29_N_2_O_6_), 328 *m/z* (C_18_H_18_N_2_O_5_), 151 *m/z* (C_8_H_9_O_3_).

#### 3.3.3. Quantification of Phenolic Compounds

Compared to H, a substantial reduction in free phenolic compounds was observed in all bioprocessed samples (Table 4). Total free phenolic compounds were almost halved by fermentation and treatment with xylanase, while that with protease reduced it by circa 30%. Glucosides of protocatechuic, tuberonic and cannabidiolic acids represented circa 30% of the total phenolic compounds identified in H. However, they were subjected to a substantial decrease due to the bioprocessing, with highest intensity when selected LAB were used (more than 80%). A small amount of the products of phenolic acids metabolism were also detected. Catechol, which was not detected in H, almost reached 3 μg/g in bioprocessed hemp, whereas 4-vinyl catechol was found only in H_F_.

*N*-caffeoyltyramine isomers constituted 20% of the overall compounds in raw hemp, reaching 61.2 μg/g, and bioprocessing treatments did not seem to significantly affect their content except for xylanase, which increased and reduced by circa 25% in both of the isomers, suggesting a possible isomerization as a consequence of the treatment.

Cannabielsoic acid isomers made up for a relevant portion of the free compound quantified in H (85.37 μg/g) and their content also decreased with all the treatments. On the contrary, cannabisin B, C and D isomers content was lower compared to the other classes of phenolic compounds. Whereas fermentation did not affect cannabisin, a significant increase in some isomers was observed in H_X_, probably due to their release from the bound phenolic fraction.

A different trend was observed for bound phenolics, for which increases of up to 20 and 60% were observed in H_P_ and H_X_, respectively (Table 4). Cannabisin B, C, D and E isomers represented most of the compounds identified, reaching 17 μg/g in H and up to 25 μg/g in bioprocessed hemp. Compared to H, a mild decrease in the two coumaric acid derivatives identified was observed while all the three flavodic acid isomers increased up to three-fold with the treatments. The concentration of *N*-caffeoyltyramine and xerocomic acid doubled in all bioprocessed samples.

### 3.4. Citotoxicity and Antioxidant Activity on Human Keratinocytes

#### 3.4.1. Cytotoxicity

**Figure 5:**
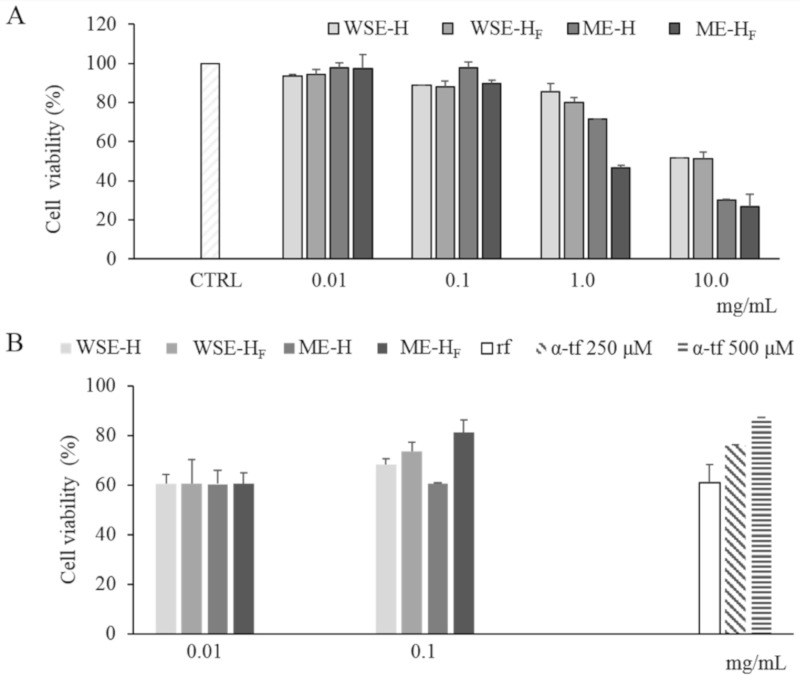
Cell viability of human keratinocytes treated with freeze-dried WSE-H and WSE-H_F_ and ME-H and ME-H_F_ at different concentrations (0.01–10 mg/mL). Cell viability of non-stressed cells (CTRL) is also reported (Panel **A**). Protective effect of freeze-dried WSE-H and WSE-H_F_ and ME-H and ME-H_F_ at concentrations of 0.01 and 0.1 mg/mL and α-tocopherol (α-tp; 250 and 500 μM) on the cell viability of human keratinocytes subjected to oxidative stress induced by hydroxide peroxide. The viability of H_2_O_2_-stressed cells incubated without antioxidant compounds (reference, rf) was also included (Panel **B**). Data are the means of three independent experiments twice analyzed. Error bars are shown.

The cytotoxicity of the freeze-dried WSE- and ME- H and H_F_ was determined on a human keratinocytes cell line. After the exposure to the extracts, the keratinocytes’ viability was determined through the MTT assay (Figure 5A). No extract showed cytotoxicity at concentrations of 0.01 and 0.1 mg/mL. Indeed, cell viability higher than 85% was found in all trials (Figure 5A). The cytotoxic effect increased according to the concentration of the extract used, regardless of the type of extract (WSE or ME) and the fermentation (H or H_F_). Nevertheless, WSEs showed a milder cytotoxic effect as compared to the corresponding MEs (Figure 5A); indeed, only MEs showed toxicity at 1 mg/mL. At 10 mg/mL, a relevant cytotoxicity was observed for all the samples (Figure 5A). No significant difference was found between the effect related to WSE-H and WSE-H_F_ treatment. On the contrary, except for 0.01 mg/mL, ME-H always led to higher cell viability than ME-H_F_ (Figure 5A).

#### 3.4.2. Protective Effect towards Oxidative-Induced Stress in Human Keratinocytes Cells

Hydrogen peroxide was used as stress treatment for keratinocytes previously cultivated in the presence of WSE- and ME- H and H_F_. In order to avoid cytotoxic effects on keratinocytes, WSE- and ME- H and H_F_ were assayed at concentrations lower than 0.1 mg/mL. Negative control (no antioxidants added) showed cell viability of 61.17 ± 7.03%, after oxidative stress. When both WSEs and MEs were used, survival was significantly higher (11–32%) than the cells of the negative control. In particular, the presence of ME-H_F_ allowed a cell survival higher (circa 7%) than the antioxidant reference α-tocopherol (250 µM) (Figure 5B). Overall, the viability of the cells after the oxidative stress was higher in presence of H_F_ than H extracts (Figure 5B).

## 4. Discussion

Initially considered as a by-product of fiber production, hempseed was rediscovered only two decades ago due to the awareness of its high nutritional value and potential functionality. The hempseed protein fraction has been reported to possess comparable nutritional value to egg white and soybean proteins because of its excellent digestibility and adequacy in essential amino acids [13]. It has also been explored for its feasibility and potential in the production of bioactive peptides with specific health-promoting activities (e.g., antioxidant) [45,46,47,48].

Besides proteins, whole hempseed is characterized by a high fiber content, mainly located in the hull [3,49], which can be responsible for several health benefits in the human body [1] and hundreds of different compounds with potential biological activity (e.g., phenolic compounds, cannabinoids, fatty acids).

Despite the high nutritional and functional potential of hempseed, it also contains some antinutritional compounds [8] which, together with the effect related to the abundance of the fiber fraction, can negatively affect the protein’s bioaccessibility and digestibility [50,51,52].

The present study investigated the effect of enzymatic treatments and fermentation on the protein and phenolic fractions of hulled seeds, evaluating the potential improvement in terms of antioxidant activity and protein digestibility.

The most active antioxidants molecules in plant matrices mainly belong to the chemical classes of phenols and peptides [53]. Commercial food-grade protease and xylanase have been used, aiming at hydrolyzing and releasing the protein fractions entrapped in the fiber structures, respectively. The protease may speed up the hydrolysis process, leading to the release of protein derivatives (mainly peptides and amino acids) with high biological activity [54,55,56] and the increase in their digestibility, whereas xylanase, through the breakdown of the insoluble fiber matrix, may expose the proteins to the endogenous and digestives enzymes, which may naturally improve the hemp functionality and digestibility [14,57]. On the other hand, studies have shown the direct and indirect effect of the lactic acid bacteria fermentation on the nutritional and functional features of hemp [12] and plant-derived food matrices in general [58,59]. Hence, *Lactiplantibacillus plantarum* 18S9 and *Leuconostoc mesenteroides* 12MM1, previously isolated from spontaneously fermented hemp flour and selected according to their pro-technological features and metabolic traits which are able to positively affect the functional and nutritional profiles of hemp [12], were also used in the study.

Hulled hempseeds harvested from cultivation under organic conditions were sterilized prior to use in order to reduce the level of epiphyte contaminants [20], milled to flour and then subjected to protease and xylanase treatments and selected lactic acid bacteria fermentation. Aiming at avoiding the fat oxidation, doughs were prepared under nitrogen stream in a dark beaker and, except for the incubation step, all the procedures were carried out under refrigerated (circa 2 °C) conditions. Optimal conditions for microbial growth occurred during the incubation, leading to an increase in microbial cell density, regardless of the type of bioprocessing applied. Due to the disinfection of the seed surface prior to use, the microbiota was mainly represented by endophytes.

Among all samples, *Enterobacteriaceae* remained stable during selected lactic acid bacteria fermentation, probably due to the inhibitory effect of the acidification [60]. As expected, when inoculated, presumptive lactic acid bacteria reached a cell density of circa 9 log10 cfu/g, leading to the highest concentrations of lactic and acetic acids. Nevertheless, increases in organic acid concentrations were found also in H_X_ and H_P_, probably due to the resident fermenting-microbiota.

Although the hempseed protein has drawn increasing attention due to its structure and nutritional properties, it has limited bioactive properties [61]. Indeed, bioactive peptides are mainly encrypted in the native protein and can be released during the hydrolysis process [1,54,56,61].

Hence, the study of proteins and their derivatives, as affected by the bioprocessing, deserves an integrated analytical approach, including the investigation of advanced proteomics analysis (2DE), peptides, and amino acid profiles.

Hempseed protein consists mainly of globulin (edestin) and albumin, with the former accounting for approximately 60–80% of the total protein content and albumin constituting the rest [48,62]. The 2DE profile of hempseed albumin and globulin fractions showed the highest abundance of proteins and polypeptides with molecular weight between 15 and 37 kDa (>50% of the total resolved). Edestin has a structure composed of six identical subunits, each consisting of an acidic (AS) and a basic (BS) subunit linked by one disulfide bond [63]). The AS is approximately 34.0 kDa and relatively homogeneous, while BS consists mainly of two subunits of about 20.0 and 18.0 kDa [62,64,65]. The total number of polypeptides (10–100 kDa) increased after protease and fermentation treatments, with the former having the most significant effect. Decreases in proteins with MW higher than 25 kDa and significant increases in polypeptides having MW lower than 25 kDa in H_F_ and H_P_ explain the proteolytic activity of both protease and selected lactic acid bacteria. However, while VeronPS led to the hydrolysis of the protein having MW higher than 37 kDa, lactic acid bacteria seemed not to be capable of such degradation. Lactic acid bacteria isolated from sourdough have already been reported to have weak proteinase activity [15]. Moreover, the highest increase in polypeptides, having 10–15 kDa in H_P_ as compared to H_F_, might be explained by the strong peptidase activity of the VeronPS [66]. A lower number of polypeptides were found in H_X_. The activity of xylanase on the hemicellulose (circa 22% of total insoluble fiber) fraction of the hull might have contributed to the release of entrapped proteins which became available to the endogenous proteinase and peptidase, leading to short peptides and free amino acids [5,14]. The incubation conditions (e.g., humidity and stable temperature) might have mimicked the germination conditions, hence allowing the activation of endogenous enzymes [67]. Indeed, significant increases in the low molecular mass peptides (<10 kDa), not retained by the 2DEand investigated through liquid chromatography, were observed as the consequence of the xylanase treatment. The same was observed for FAA. In this case, the release of soluble compounds from the fibrous cellular compartments should also be hypothesized [14]. Nevertheless, the most intense proteolysis of the native proteins was found when VeronPS was used [66], contributing to the highest concentration of peptides. The highest content of TFAA was achieved during lactic acid bacteria fermentation due to the combination of the endogenous proteolytic enzymes and microbial peptidases [68]. Overall, lactic acid bacteria activate the endogenous proteinase, through acidification, leading to the release of medium-sized polypeptides which are subjected to microbial peptidase activities [68]. The highest value of in vitro protein digestibility (79%) in fermented hempseed corroborates the above hypothesis.

According to the in vitro analysis, H_F_ exerted the strongest antioxidant activity in both ME and WSE extracts and compared to enzymatic-treated samples (60 and 72% vs. 50%). Microbial fermentation has widely been recognized as one of the major and economically most convenient processes, with lactic acid bacteria being used as cell factories, to produce bioactive peptides from a variety of food protein sources [9]. Short peptides, displaying antioxidant activity, are often hidden in a latent state within the primary sequences of food proteins; indeed, enzymatic proteolysis is required for their release [69].

The water-salt soluble extract of the fermented hemp was, therefore, fractionated through RP-FPLC and the peptides of the most active fraction (antioxidant activity > 50%) purified and identified by (nano-LC-ESI-MS/MS. The activity of the WSE was affected up to 20% by the enzymatic digestion. With only one exception (length, 24 amino acid; molecular weight 2638 Da), the mixtures contained small peptides (9–15 amino acid) with a molecular weight in the range of 800 −1500 Da. Overall, it has been reported that the antioxidative activity of a peptide mixture is an integrative effect of the actions rather than due to their individual actions [70]. Antioxidant peptides usually contain 5–16 amino acid residues [71], with those having a molecular weight of 500–1500 Da being the most active [72]. All the sequences were encrypted in native *C. sativa* proteins and characterized by high content of hydrophobic (up to 66%) and branched-chain amino acids (up to 28%). Overall, the antioxidant potential of plant-peptides is partly ascribed to their amino acid composition. Previously identified hemp peptides [46] showing strong anti-oxidative and -hypertensive actions were characterized by the presence of substantial amounts of hydrophobic, branched-chain and aromatic amino acid residues [73].

As already reported, hempseed is rich in phenolic compounds, tocopherols, carotenoids, and phytosterols with a strong antioxidant potential [6,74]. However, the degradation of phenolic acid esters, glycosides, and tannins, leading to the release of more active derivatives and the biochemical changes in the products (mainly acidic conditions), have been reported to positively affect the solubility and extractability [59]. Hence, the effect of bioprocessing on hempseed phenolic compounds has been investigated by means of their quantification and identification.

Even though the total free phenolic compounds identified decreased after the bioprocessing treatments, a relevant increase in the antioxidant activity in the methanolic extract of bioprocessed hemp was observed, especially in fermented hemp. Although it might seem contradictory, the reduction mostly affected glucosides and their phenolic acids, which led to the development of other compounds. Many lactic acid bacteria are known to metabolize hydroxycinnamic acids, as a defense mechanism, decarboxylating and/or reducing them to the corresponding vinyl or ethyl derivatives with a higher antioxidant activity than their precursors [75]. Among these, 4-vinyl catechol and 4-vinyl phenol, products of the decarboxylation of caffeic and *p*-coumaric acids [39], respectively, were detected as having a higher content in fermented hemp. 3-(3-dihydroxyphenyl)propionic acids, also described as a product of *m*-coumaric acid degradation [39], compared to raw hemp, increased up to five times during fermentation (Table 4), and was also shown to have in vivo vasodilatory activity in both normotensive and spontaneously hypertensive rats, leading to a blood pressure decrease [76]. It should also be considered that these metabolites are volatile and the extraction protocol, including the rotary evaporation, could interfere with their correct quantification.

Among the phenolic compounds identified, cannabisin C and D, *N*-*trans*-caffeoyltyramine, and *N*-*trans*-feryroyltyramine, which also increased during bioprocessing, are known for their powerful DPPH and ABTS radical-scavenging activity, comparable to reference antioxidants [44]. Fluctuations of the phenolic compounds in enzyme-treated hemp are probably due to a combination of the endogenous microbiota and the enzyme activity itself. As a matter of fact, protein–phenolic complexes in plant matrices, including hemp, have been reported [77]. It is likely that their release is a consequence of VeronPS activity on the protein fraction.

While the most relevant changes observed in free phenolic profile were due to lactic acid bacteria bioconversion pathways of phenolic acids, the bound phenolic profile was mainly affected by enzymatic treatments. Just as reported for brewers’ spent grain treated with Depol 761P [19], the extractability of bound phenolics increased by up to 60% when the same xylanase was used for hemp. The intense activity of the enzyme most likely weakened phenolic compound bonds with the lignocellulosic material, facilitating the hydrolysis and the extraction process.

Since in vitro assays are only considered as predictive tools for the antioxidant activity in vivo and testing a substance directly on animals or human is not an easy approach, different methods comprising cellular models were recently developed [78]. In this study, a well-known human keratinocyte cell line was subjected to oxidative-induced stress with peroxide hydroxide and subjected to the MTT assay, which allows the cell survival estimation after stress exposure. The test was performed in the presence of the extracts obtained from ME and WSE-fermented hemp, aiming at evaluating their protective effect against oxidative stress. Compared to the raw hempseed, fermentation conferred to the matrix a relevant protective activity in all the tested conditions, especially when the ME was used. Studies have already demonstrated that the antioxidant capacity of hempseed is due to the combination of phenolic compounds rather than to the tocopherols.

## 5. Conclusions

This study demonstrated the suitability of hemp seed to be used as functional ingredient when subjected to biotransformation. Although modification occurred also as a consequence of the enzymatic treatments, fermentation with selected lactic acid bacteria caused changes, not only affecting the biochemical characteristics of the matrix, but also providing the most promising potential in terms of nutritional and functional properties. Indeed, the intense proteolysis led to high peptide and free amino acid concentrations, which positively influenced the in vitro protein digestibility of the hemp, reaching a value of 79%. High IVPD, in turn, might improve the hemp as well as the hemp-food protein quality available for digestion and assimilation by the human body. The changes that occurred during fermentation also led to an increase in the antioxidant property, as determined in vitro (up to 70%) and on the human keratinocyte cell line. This property is of major importance for food stability against oxidation after storage and as a bioactivity for human health, an interesting feature which needs further in-depth study.

## Figures and Tables

**Table 1:** Microbiological and biochemical characteristics of raw and bioprocessed hemp. H, mixture of hemp flour and water (ratio 1:1); H_X_, mixture of hemp flour and water (ratio 1:1) treated with xylanase Depol 761P (1% wt/wt of fiber); H_P_, mixture of hemp flour and water (ratio 1:1) treated with protease Veron PS (2.5% wt/wt of protein); H_F_, mixture of hemp flour and water (ratio 1:1) fermented by *Lactiplantibacillus plantarum* 18S9 and *Leuconostoc mesenteroides* 12MM1 (ratio 1:1, final cell density of circa 7 log10cfu/g). All treated samples were incubated at 30 °C for 24 h.

	H	H_X_	H_P_	H_F_
***Microbiological characteristics***				
TMB (log10 cfu/g)	3.5 ± 0.2 ^c^	5.8 ± 0.1 ^b^	5.8 ± 0.1 ^b^	9.0 ± 0.2 ^a^
LAB (log10 cfu/g)	3.5 ± 0.3 ^c^	4.7 ± 0.2 ^b^	4.7 ± 0.1 ^b^	9.0 ± 0.2 ^a^
Yeasts (log10 cfu/g)	3.1 ± 0.2 ^b^	4.1 ± 0.3 ^a^	4.0 ± 0.2 ^a^	3.9 ±0.1 ^a^
Molds (log10 cfu/g)	2.5 ± 0.2 ^b^	3.4 ± 0.3 ^a^	3.2 ± 0.2 ^a^	3.1 ±0.1 ^a^
*Enterobacteriacae* (log10 cfu/g)	4.7 ± 0.1 ^b^	5.9 ± 0.2 ^a^	5.7 ± 0.2 ^a^	4.6 ± 0.1 ^b^
***Biochemical properties***				
pH	6.60 ± 0.03 ^a^	5.61 ± 0.02 ^c^	5.77 ± 0.03 ^b^	5.21 ± 0.04 ^d^
Lactic acid (mmol/kg)	1.79 ± 0.24 ^d^	50.18 ± 1.2 ^b^	40.93 ± 1.1 ^c^	88.25 ± 2.2 ^a^
Acetic acid (mmol/kg)	3.16 ± 0.31 ^d^	18.46 ± 0.94 ^b^	13.40 ± 1.11 ^c^	37.67 ± 2.4 ^a^
TFAA (mg/kg)	1656 ± 23 ^d^	4843 ± 27 ^c^	5115 ± 31 ^b^	5198 ± 26 ^a^
Peptides (mg/kg)	259.84 ± 10 ^d^	338.13 ± 11 ^c^	371.31 ± 23 ^a^	350.92 ± 15 ^b^

TMB, total mesophilic bacteria; LAB, lactic acid bacteria; TFAA, total free amino acids. Data are the means of three independent experiments ± standard deviations (*n* = 3). ^a–d^ Values in the same row with different superscript letters differ significantly (*p* < 0.05).

**Table 2:** List of the peptides identified in the partially purified peptide fraction obtained from the fermented hemp through RP-FPLC. Fermentation was carried out by *Lactiplantibacillus plantarum* 18S9 and *Leuconostoc mesenteroides* 12MM1 (ratio 1:1, final cell density of circa 10^7^ cfu/g) at 30 °C for 24 h.

Fraction	Sequence	Weight (Da)	Length (aa)	Net Charge	Hydrophobic-AA (%)	BCAA * (%)	NCBI Accession n° (Protein)	Fragment
1	ALASIGKATR	986.6	10	2	60	20	XP_030502118.1 (nephrocystin-3)	233–242
2	IGQSHPQALMYPLLVACKSISNLR	2638.4	24	3	52	28	XP_030508478.1 (Serine/threonine-protein kinase TOR)	1863–1886
5	AQVSVGGGR	829.4	9	2	66	22	XP_030499964.1 (edestin 3)	119–127
7	AIENGAVSVSEPEEK	1557.7	15	2	44	19	XP_030503904.1 (uncharacterized protein)	99–113
	DLQIIAPSR	1011.5	9	2	55	33	SNQ45155.1 (edestin 3)	286–194

* BCAA, branched-chain amino acids.

**Table 3:** Phenolic compounds identified in raw and bioprocessed hemp by HPLC-DAD-ESI-QTOF-MS. H, untreated hemp dough; H_X_, hemp dough treated with xylanase Depol 761P (1% wt/wt of fiber); H_P_, hemp dough treated with protease VeronPS (2.5% wt/wt of protein); H_F_, hemp dough fermented by *Lactiplantibacillus plantarum* 18S9 and *Leuconostoc mesenteroides* 12MM1 (ratio 1:1, final cell density of circa 7 log10 cfu/g). All treated samples were incubated at 30 °C for 24 h.

N°	Polar Compound	RT (min)	Molecular Formula	*m/z* Expected	*m/z* Calculated	Error (ppm)	Score (%)	Fragments (MS^2^)
***Free compounds***								
1	Protocatechuic acid-glucoside isomer 1	2.946	C_13_H_16_O_9_	315.0703	315.0716	−4.1	99.78	
2	Protocatechuic acid-glucoside isomer 2	3.281	C_13_H_16_O_9_	315.0713	315.0716	−1	98.96	
3	Catechol	4.560	C_6_H_6_O_2_	109.0288	109.0290	−1.8	92.24	
4	4-vinyl catechol	4.730	C_8_H_8_O_2_	135.0435	135.0446	−8.1	87.98	
5	Tuberonic acid glucoside isomer 1	7.939	C_18_H_28_O_9_	387.1649	387.1655	−1.5	94.94	
6	4-vinyl guaiacol	8.178	C_9_H_10_O_2_	149.0603	149.1770	−14.1	79.88	
7	Tuberonic acid glucoside isomer 2	8.295	C_18_H_28_O_9_	387.1642	387.1655	−3.4	94.94	
8	3-(3-Hydroxyphenyl)propionic acid	8.530	C_9_H_10_O_3_	165.0543	165.0552	−5.5	92.86	
9	4-vinyl phenol	8.543	C_8_H_8_O_0_	119.0489	119.0497	−6.7	81.46	
10	Tuberonic acid glucoside isomer 3	8.783	C_18_H_28_O_9_	387.1646	387.1655	−2.3	94.94	
11	Tuberonic acid glucoside isomer 4	8.882	C_18_H_28_O_9_	387.1656	387.1655	0.3	94.94	
12	Tuberonic acid glucoside isomer 5	9.797	C_18_H_28_O_9_	387.1652	387.1655	−0.8	94.94	
13	*N*-caffeoyltyramine	12.3	C_17_H_17_NO_4_	298.1074	298.1079	−1.7	99.54	
14	Luteolin	12.63	C_15_H_9_O_6_	285.0396	285.0396	−1.1	98.68	
15	*N*-caffeoyltyramine	13.81	C_17_H_17_NO_4_	298.1074	298.1079	−1.7	99.54	
16	Cannabisin B isomer 1	15.98	C_34_H_32_N_2_O_8_	595.2066	595.2080	−2.4	78.54	432.1450, 322.1068, 269.0814
17	Cannabisin B isomer 2	16.18	C_34_H_32_N_2_O_8_	595.2076	595.2080	−0.7	78.54	432.1450, 322.1068, 269.0814
18	Cannabisin B isomer 3	16.81	C_34_H_32_N_2_O_8_	595.2079	595.2080	−0.1	78.54	
19	Cannabisin B isomer 4	17.3	C_34_H_32_N_2_O_8_	595.209	595.2080	1.7	78.54	432.1450, 322.1068, 269.0814
20	Cannabisin C isomer 1	17.61	C_35_H_34_N_2_O_8_	609.2242	609.2237	0.8	76.51	
21	Cannabisin C isomer 2	17.87	C_35_H_34_N_2_O_8_	609.2249	609.2237	2	76.51	446.1611, 283.0970, 268.0736
22	Cannabisin C isomer 3	18.38	C_35_H_34_N_2_O_8_	609.2243	609.2237	1	76.51	
23	Cannabisin D isomer 1	19.04	C_36_H_36_N_2_O_8_	623.2393	623.2393	0	70.77	460.1760, 445.1523
24	Cannabisin D isomer 2	19.35	C_36_H_36_N_2_O_8_	623.2386	623.2393	−1.1	70.77	460.1760, 445.1523
25	Cannabisin D isomer 3	20.73	C_36_H_36_N_2_O_8_	623.2386	623.2393	−1.1	70.77	
26	Trihydroxyoctadeceno ic acid	21.11	C_18_H_34_O_5_	329.2320	329.2328	−2.4	98.65	
27	Trihydroxyoctadeceno ic acid	21.21	C_18_H_34_O_5_	329.2319	329.2328	−0.9	98.65	
28	Trihydroxyoctadeceno ic acid	21.32	C_18_H_34_O_5_	329.2326	329.2328	−0.2	98.65	
29	Cannabisin D isomer 4	21.57	C_36_H_36_N_2_O_8_	623.2393	623.2393	0	70.77	460.1760, 445.1523
30	Cannabisin D isomer 5	21.74	C_36_H_36_N_2_O_8_	623.2409	623.2393	2.6	70.77	
31	Cannabidiolic acid glucoside	23.35	C_28_H_40_O_9_	519.2593	519.2594	−0.2	56.29	373.1255
32	Cannabielsoic acid	23.61	C_22_H_30_O_5_	373.2012	373.2015	−0.8	97.08	
33	Cannabielsoic acid	23.65	C_22_H_30_O_5_	373.2014	373.2015	−0.3	97.08	
34	Cannabielsoic acid	23.86	C_22_H_30_O_5_	373.2008	373.2015	−0.7	97.08	
35	Cannabielsoic acid	23.97	C_22_H_30_O_5_	373.2015	373.2015	0	97.08	
36	Cannabielsoic acid	24.12	C_22_H_30_O_5_	373.2008	373.2015	−0.7	97.08	
37	Cannabielsoic acid	24.41	C_22_H_30_O_5_	373.2013	373.2015	−0.5	97.08	
38	Cannabielsoic acid	24.92	C_22_H_30_O_5_	373.2025	373.2015	2.7	97.08	
39	THCA derivative	25.18	C_46_H_58_O_8_	737.4056	737.4053	0.4	96.53	
40	Fatty acid C18:3	25.77	C_18_H_30_O_2_	277.2158	277.2168	−3.6	99.75	
41	Fatty acid C18:3	25.806	C_18_H_30_O_2_	277.2164	277.2168	−1.4	97.64	
42	Fatty acid C18:2	26.117	C_18_H_32_O_2_	279.232	279.2324	−1.14	91.75	
***Bound compounds***								
1	Citric acid	0659	C_6_H_8_O_7_	191.0182	191.0192	−5.2	79.55	111.0079
2	Citric acid	0.882	C_6_H_8_O_8_	191.0185	191.0192	−3.7	79.55	111.0079
3	Protocatechuic acid derivative	3.231	C_15_H_8_O_9_	331.0024	331.256	1.2	98.65	153.0186, 109.0287
4	Protocatechuic acid glucoside	4.357	C_13_H_16_O_9_	315.0654	315.0716	−19.7	70.77	
5	Salicylic acid derivative	4.849	C_15_H_8_O_7_	299.0131	299.0192	−17.7	98.65	137.0233
6	Benzoic acid aldehyde	6.566	C_7_H_6_O_2_	121.0283	121.029	−5.8	79.27	92.0246
7	Catechin	6.619	C_15_H_14_O_6_	289.0702	289.0712	−3.5	75.65	
8	Xerocomic acid	8.659	C_18_H_12_O_8_	355.0442	355.0454	−3.1	86.29	337.0336, 311.0544, 267.0651
9	*trans p*-Coumaric acid	9.24	C_9_H_8_O_3_	163.0388	163.0395	−4.3	98.97	119.0485
10	*cis p*-Coumaric acid	9.47	C_9_H_8_O_3_	163.0386	163.0395	−5.5	96.28	119.0485
11	Coumaric acid derivative	9.84	C_18_H_16_O_6_	327.0858	327.0869	−4.3	95.71	163.039, 119.0481
12	Coumaric acid derivative	10.36	C_18_H_16_O_6_	327.0858	327.0869	−4.3	95.71	163.039, 119.0481
13	Flavodic acid	10.87	C_19_H_14_O_8_	369.0589	369.061	−5.7	96.08	337.0344, 265.0555
14	Flavodic acid	12.151	C_19_H_14_O_8_	369.0589	369.061	−2.4	95.98	337.0344. 265.0493
15	Flavodic acid	12.8	C_19_H_14_O_8_	369.0589	369.061	−2.4	95.98	
16	*N*-caffeoyltyramine	13.87	C_17_H_17_NO_4_	298.1096	298.1079	−5	98.29	178.0506, 135.0434
17	Flavodic acid derivative	14.11	C_20_H_16_O_8_	383.0779	383.0767	3.1	95.56	337.0344, 351.0500, 263.0555
18	Cannabisin B isomer 1	16.06	C_34_H_32_N_2_O_8_	595.2089	595.208	−1.3	97.08	
19	Cannabisin B isomer 2	16.23	C_34_H_32_N_2_O_8_	595.2089	595.208	1.5	97.08	432.1450, 322.1068, 269.0814
20	*N*-*trans*-feryroyltyramine	16.544	C_18_H_19_NO_4_	312.1225	312.1236	−3.5	92.64	190.0481, 135.0426, 178.0484
21	Cannabisin B isomer 3	16.853	C_34_H_32_N_2_O_8_	595.2092	595.208	2	97.08	432.1450, 322.1068, 269.0814
22	Cannabisin B isomer 4	17.24	C_34_H_32_N_2_O_8_	595.2092	595.208	1	97.08	
23	Cannabisin B isomer 5	17.366	C_34_H_32_N_2_O_8_	595.2072	595.208	−1.3	97.08	485.1701, 432.1450, 322.1068, 269.0814
24	Cannabisin C isomer 1	17.65	C_35_H_34_N_2_O_8_	609.2229	609.2237	−1.3	96.22	
25	Cannabisin B isomer 6	17.773	C_34_H_32_N_2_O_8_	595.2065	595.208	−2.5	97.08	322.1068, 269.0814
26	Cannabisin C isomer 2	17.93	C_35_H_34_N_2_O_8_	609.2229	609.2237	−1.1	96.22	
27	Cannabisin C isomer 3	18.12	C_35_H_34_N_2_O_8_	609.2229	609.2237	−1.2	96.22	
28	Cannabisin C isomer 4	18.42	C_35_H_34_N_2_O_8_	609.2231	609.2237	−1	96.22	446.1611, 283.0970, 268.0736
29	Cannabisin D isomer 1	19.06	C_36_H_36_N_2_O_8_	623.2392	623.2393	−0.2	98.39	460.1760, 445.1523
30	Cannabisin B isomer 7	19.19	C_34_H_32_N_2_O_8_	595.2065	595.208	−2.5	97.08	432.1450, 322.1068, 269.0814
31	Cannabisin B isomer 8	19.55	C_34_H_32_N_2_O_8_	595.2065	595.208	−2.5	97.08	432.1450, 322.1068, 269.0814
32	Cannabisin D isomer 2	19.378	C_36_H_36_N_2_O_8_	623.2402	623.2393	1.4	98.39	
33	Cannabisin E isomer 1	19.633	C_36_H_38_N_2_O_9_	641.2493	641.2499	1.6	97.81	489.2026, 328.1187, 151.0380
34	Cannabisin E isomer 2	19.92	C_36_H_38_N_2_O_9_	641.2509	641.2499	1.6	97.81	489.2026, 328.1187, 151.0380
35	Cannabisin C isomer 5	20.156	C_35_H_34_N_2_O_8_	609.2242	609.2237	0.8	96.22	
36	Cannabisin D isomer 3	20.21	C_36_H_36_N_2_O_8_	623.2398	623.2393	0.8	98.39	
37	Cannabisin C isomer 6	20.52	C_35_H_34_N_2_O_8_	609.223	609.2237	−1.1	96.22	
38	Cannabisin D isomer 4	21.579	C36H_36_N_2_O_8_	623.2405	623.2393	1.9	98.39	
39	THCA derivative	25.18	C_46_H_58_O_8_	737.4056	737.4053	0.4	96.53	

**Table 4:** Quantification (μg/g d.w.) of the phenolic compounds identified in raw and bioprocessed hemp. H, untreated hemp dough; H_X_, hemp dough treated with xylanase Depol 761P (1% wt/wt of fiber); H_P_, hemp dough treated with protease VeronPS (2.5% wt/wt of protein); H_F_, hemp dough fermented by *Lactiplantibacillus plantarum* 18S9 and *Leuconostoc mesenteroides* 12MM1 (ratio 1:1, final cell density of circa 7 log10 cfu/g). All treated samples were incubated at 30 °C for 24 h.

	H	H_X_	H_P_	H_F_
***Free phenolics***				
Protocatechuic acid-glucoside	27.0 ± 0.1 ^a^	3.7 ± 0.2 ^c^	6.1 ± 0.1 ^b^	n.d
Protocatechuic acid-glucoside	33.5 ± 0.8 ^a^	4.99 ± 0.06 ^c^	8.66 ± 0.07 ^b^	5.9 ± 0.6 ^c^
Catechol	n.d.	2.94 ± 0.08 ^a^	2.95 ± 0.01 ^a^	2.60 ± 0.09 ^b^
4-vinyl catechol	n.d.	n.d.	n.d.	4.01 ± 0.01
Tuberonic acid glucoside	3.43 ± 0.05 ^a^	2.26 ± 0.03 ^b^	2.30 ± 0.11 ^b^	1.92 ± 0.01 ^c^
4-vinyl guaiacol	2.14 ± 0.06 ^a^	n.d.	1.98 ± 0.01 ^b^	1.88 ± 0.01 ^b^
Tuberonic acid glucoside	3.09 ± 0.06 ^a^	1.99 ± 0.02 ^b^	2.08 ± 0.06 ^b^	1.89 ± 0.01 ^b^
3-(3-Hydroxyphenyl) propionic acid	1.96 ± 0.02 ^c^	3.95 ± 0.07 ^b^	3.18 ± 0.08 ^b^	10.48 ± 0.04 ^a^
4-vinyl phenol	1.87 ± 0.02 ^c^	2.53 ± 0.00 ^b^	2.28 ± 0.06 ^b^	4.70 ± 0.06 ^a^
Tuberonic acid glucoside	3.37 ± 0.01 ^a^	1.94 ± 0.01 ^b^	2.13 ± 0.06 ^b^	1.88 ± 0.01 ^c^
Tuberonic acid glucoside	19.4 ± 0.2 ^a^	3.32 ± 0.01 ^c^	5.6 ± 0.3 ^b^	2.67 ± 0.02 ^d^
Tuberonic acid glucoside	2.23 ± 0.03 ^a^	2.04 ± 0.04 ^b^	2.08 ± 0.06 ^b^	1.81 ± 0.01 ^c^
*N*-caffeoyltyramine	32.2 ± 0.5 ^a^	24 ± 2 ^b^	33 ± 2 ^a^	31.2 ± 0.6 ^a^
Luteolin	<LOQ	<LOQ	<LOQ	<LOQ
*N*-caffeoyltyramine	29 ± 2 ^b^	36 ± 1 ^a^	34 ± 4 ^a^	26.2 ± 0.9 ^b^
Cannabisin B	0.81 ± 0.05 ^b^	1.02 ± 0.06 ^a^	1.0 ± 0.1 ^a^	0.86 ± 0.05 ^b^
Cannabisin B	20.4 ± 0.4 ^a^	20 ± 2 ^a^	21.0 ± 0.1 ^a^	19.6 ± 0.5 ^a^
Cannabisin B	1.16 ± 0.02 ^b^	1.51 ± 0.09 ^a^	1.4 ± 0.2 ^a^	0.98 ± 0.03 ^c^
Cannabisin B	0.92 ± 0.09 ^a^	1.11 ± 0.02 ^a^	1.1 ± 0.2 ^a^	0.61 ± 0.08 ^b^
Cannabisin C	6.3 ± 0.1 ^ab^	6.72 ± 0.06 ^a^	6.5 ± 0.2 ^a^	5.7 ± 0.2 ^b^
Cannabisin C	5.60 ± 0.02 ^a^	5.7 ± 0.4 ^a^	5.9 ± 0.3 ^a^	5.0 ± 0.4 ^a^
Cannabisin C	3.0 ± 0.2 ^ab^	3.3 ± 0.3 ^a^	3.7 ± 0.3 ^a^	2.61 ± 0.03 ^b^
Cannabisin D	4.4 ± 0.1 ^a^	4.3 ± 0.5 ^a^	4.32 ± 0.05 ^a^	4.0 ± 0.3 ^a^
Cannabisin D	0.93 ± 0.07 ^a^	1.3 ± 0.3 ^a^	1.4 ± 0.6 ^a^	0.38 ± 0.06 ^b^
Cannabisin D	4.80 ± 0.09 ^b^	4.2 ± 0.4 ^b^	6.2 ± 0.3 ^a^	5.9 ± 0.2 ^a^
Cannabisin D	3.1 ± 0.1 ^b^	5.1 ± 0.2 ^a^	4.5 ± 0.5 ^a^	2.1 ± 0.2 ^c^
Cannabisin D	3.3 ± 0.2 ^a^	4.1 ± 0.6 ^a^	3.7 ± 0.5 ^a^	2.37 ± 0.04 ^b^
Cannabidiolic acid glucoside	7.62 ± 0.01 ^a^	3.3 ± 0.2 ^b^	4.0 ± 0.2 ^b^	3.77 ± 0.05 ^b^
Cannabielsoic acid	13.3 ± 0.2 ^a^	2.9 ± 0.2 ^d^	4.35 ± 0.01 ^b^	3.90 ± 0.09 ^c^
Cannabielsoic acid	9.86 ± 0.01 ^a^	2.7 ± 0.1 ^d^	3.5 ± 0.1 ^b^	3.24 ± 0.01 ^c^
Cannabielsoic acid	4.63 ± 0.04 ^a^	1.90 ± 0.01 ^c^	2.06 ± 0.01 ^b^	2.05 ± 0.04 ^b^
Cannabielsoic acid	6.81 ± 0.02 ^a^	1.96 ± 0.03 ^d^	2.40 ± 0.01 ^b^	2.27 ± 0.01 ^c^
Cannabielsoic acid	4.57 ± 0.02 ^a^	1.96 ± 0.03 ^d^	2.64 ± 0.09 ^b^	2.22 ± 0.01 ^c^
Cannabielsoic acid	16.2 ± 0.1 ^a^	3.0 ± 0.1 ^b^	3.8 ± 0.1 ^b^	3.42 ± 0.07 ^b^
Cannabielsoic acid	30 ± 2 ^a^	3.5 ± 0.4 ^c^	4.8 ± 0.4 ^b^	4.6 ± 0.2 ^b^
**Total**	307 ± 6 ^a^	170 ± 6 ^c^	194 ± 4 ^b^	173 ± 2 ^c^
***Bound phenolics***				
Salicylic acid derivative	14.4 ± 0.5 ^c^	59 ± 2 ^a^	32 ± 2 ^b^	7.2 ± 0.5 ^d^
Benzoic acid aldehyde	26 ± 3 ^a^	7.20 ± 0.20 ^c^	6.60 ± 0.2 ^c^	13 ± 1 ^b^
Xerocomic acid	1.37 ± 0.01 ^c^	3.60 ± 0.20 ^b^	3.60 ± 0.1 ^b^	4.10 ± 0.11 ^a^
*trans p*-Coumaric acid	0.98 ± 0.03 ^a^	0.91 ± 0.02 ^a^	0.98 ± 0.02 ^a^	1.02 ± 0.06 ^a^
*cis p*-Coumaric acid	0.56 ± 0.02 ^a^	0.49 ± 0.01 ^b^	0.54 ± 0.01 ^a^	0.56 ± 0.01 ^a^
Coumaric acid derivative	0.84 ± 0.05 ^a^	0.50 ± 0.01 ^c^	0.68 ± 0.01 ^b^	0.90 ± 0.01 ^a^
Coumaric acid derivative	1.89 ± 0.07 ^a^	0.95 ± 0.07 ^c^	0.85 ± 0.02 ^c^	1.47 ± 0.02 ^b^
Flavodic acid	0.37 ± 0.02 ^a^	0.30 ± 0.02 ^b^	0.32 ± 0.01 ^b^	0.43 ± 0.01 ^a^
Flavodic acid	0.80 ± 0.10 ^c^	2.45 ± 0.06 ^a^	2.03 ± 0.08 ^b^	2.1 ± 0.10 ^b^
Flavodic acid	0.48 ± 0.01 ^c^	1.52 ± 0.10 ^a^	1.14 ± 0.04 ^b^	1.21 ± 0.04 ^b^
*N*-caffeoyltyramine	11.04 ± 0.02 ^c^	24 ± 2 ^a^	21.0 ± 0.6 ^b^	23.3 ± 0.9 ^a^
Flavodic acid derivative	0.70 ± 0.03 ^ab^	0.81 ± 0.07 ^a^	0.61 ± 0.04 ^b^	0.75 ± 0.04 ^a^
Flavodic acid derivative	0.23 ± 0.02 ^a^	0.26 ± 0.03 ^a^	0.16 ± 0.01 ^b^	0.24 ± 0.01 ^a^
Cannabisin B isomer 1	0.17 ± 0.01 ^c^	0.58 ± 0.02 ^a^	0.53 ± 0.01 ^a^	0.26 ± 0.02 ^b^
Cannabisin B isomer 2	2.01 ± 0.21 ^b^	4.4 ± 0.50 ^a^	3.6 ± 0.3 ^a^	2.4 ± 0.2 ^b^
*N*-*trans*-feryroyltyramine	3.51 ± 0.40 ^a^	3.21 ± 0.00 ^a^	3.11 ± 0.04 ^a^	3.5 ± 0.1 ^a^
Cannabisin B isomer 3	1.12 ± 0.10 ^c^	1.56 ± 0.01 ^a^	1.37 ± 0.07 ^b^	1.55 ± 0.05 ^a^
Cannabisin B isomer 4	0.14 ± 0.01 ^d^	0.74 ± 0.09 ^a^	0.49 ± 0.03 ^b^	0.32 ± 0.02 ^c^
Cannabisin B isomer 5	0.61 ± 0.01 ^c^	1.33 ± 0.03 ^a^	1.12 ± 0.08 ^b^	0.9 ± 0.1 ^b^
Cannabisin C isomer 1	1.17 ± 0.07 ^b^	2.41 ± 0.10 ^a^	2.22 ± 0.20 ^a^	2.30 ± 0.05 ^a^
Cannabisin B isomer 6	0.16 ± 0.00 ^c^	0.33 ± 0.02 ^a^	0.28 ± 0.02 ^a^	0.23 ± 0.02 ^b^
Cannabisin C isomer 2	0.37 ± 0.03 ^a^	1.70 ± 0.10 ^a^	1.60 ± 0.05 ^a^	1.26 ± 0.03 ^b^
Cannabisin C isomer 3	0.53 ± 0.04 ^a^	0.59 ± 0.04 ^a^	0.53 ± 0.03 ^a^	0.54 ± 0.00 ^a^
Cannabisin C isomer 4	2.21 ± 0.10 ^b^	2.89 ± 0.11 ^a^	2.63 ± 0.07 ^a^	2.72 ± 0.10 ^a^
Cannabisin D isomer 1	1.92 ± 0.02 ^a^	1.71 ± 0.05 ^b^	1.64 ± 0.10 ^b^	1.68 ± 0.04 ^b^
Cannabisin B isomer 7	0.36 ± 0.02 ^c^	1.14 ± 0.04 ^a^	1.00 ± 0.04 ^ab^	0.85 ± 0.02 ^b^
Cannabisin B isomer 8	0.15 ± 0.01 ^c^	0.47 ± 0.02 ^a^	0.45 ± 0.02 ^a^	0.26 ± 0.01 ^b^
Cannabisin D isomer 2	1.03 ± 0.05 ^a^	1.01 ± 0.01 ^a^	0.99 ± 0.03 ^a^	0.98 ± 0.02 ^a^
Cannabisin E isomer 1	0.82 ± 0.01 ^a^	0.61 ± 0.05 ^b^	0.68 ± 0.01 ^b^	0.56 ± 0.01 ^c^
Cannabisin E isomer 2	0.41 ± 0.02 ^a^	0.39 ± 0.03 ^a^	0.38 ± 0.03 ^a^	0.32 ± 0.03 ^a^
Cannabisin C isomer 5	0.31 ± 0.01 ^a^	0.32 ± 0.02 ^a^	0.28 ± 0.01 ^a^	0.25 ± 0.02 ^b^
Cannabisin D isomer 3	1.62 ± 0.10 ^a^	1.29 ± 0.05 ^b^	1.29 ± 0.04 ^b^	1.3 ± 0.10 ^b^
Cannabisin C isomer 6	0.39 ± 0.01 ^b^	0.44 ± 0.04 ^a^	0.36 ± 0.01 ^b^	0.30 ± 0.03 ^c^
Cannabisin D isomer 4	0.34 ± 0.04 ^b^	0.45 ± 0.00 ^a^	0.40 ± 0.04 ^a^	0.15 ± 0.02 ^c^
Cannabisin D isomer 5	1.12 ± 0.09 ^b^	1.28 ± 0.09 ^a^	1.37 ± 0.07 ^a^	0.79 ± 0.05 ^c^
**Total**	81 ± 5 ^c^	130 ± 6 ^a^	97 ± 5 ^b^	80 ± 4 ^c^

Data are the means of three independent experiments analyzed twice. ^a–d^ Values in the same row with different superscript letters differ significantly (*p* < 0.05). n.d., not detected; LOQ, limit of quantification.

## Supplementary Materials

The following are available online at https://www.mdpi.com/2076-3921/9/12/1258/s1, Table S1: Estimated molecular weight range (kDa) and pI of polypeptides found in raw and bioprocessed hemp: H, mixture of hemp flour and water (ratio 1:1); H_X_, mixture of hemp flour and water (ratio 1:1) treated with xylanase Depol 761P (1% wt/wt of fiber); H_P_, mixture of hemp flour and water (ratio 1:1) treated with protease Veron PS (2.5% wt/wt of protein); H_F_, mixture of hemp flour and water (ratio 1:1) fermented by *Lactiplantibacillus plantarum* 18S9 and *Leuconostoc mesenteroides* 12MM1 (ratio 1:1, final cell density of circa 10^7^ cfu/g). All treated samples were incubated at 30 °C for 24 h.
