# Supplementary material for: Impact of Enzymatic and Microbial Bioprocessing on Antioxidant Properties of Hemp (Cannabis sativa L.)
Source: Antioxidants (Basel). 2020 Dec 10;9(12):1258. doi: 10.3390/antiox9121258 (PMC7763576; doi:10.3390/antiox9121258)
Supplement: Supplementary file 1 [file antioxidants-09-01258-s001.pdf]

Supplementary Table 1. Estimated molecular weight range (kDa) and pI of polypeptides found in raw and bioprocessed hemp: H, mixture of hemp flour and water (ratio 1:1); H_X, mixture of hemp flour and water (ratio 1:1) treated with xylanase Depol 761P (1% wt/wt of fiber); H_P, mixture of hemp flour and water (ratio 1:1) treated with protease Veron PS (2.5% wt/wt of protein); H_F, mixture of hemp flour and water (ratio 1:1) fermented by *Lactiplantibacillus plantarum* 18S and *Leuconostoc mesenteroides* 12MM1 (ratio 1:1, final cell density of *circa* 10^7 cfu/g). All treated samples were incubated at 30° C for 24 h.

MW (kDa)	pI range	H	H_X	H_P	H_F
> 37 kDa	3 - 5.4	0	2	1	1
	5.4 - 7.3	7	1	2	8
	7.3 -10	0	0	3	1
20-37 kDa	3 - 5.4	2	3	1	4
	5.4 - 7.3	38	25	31	28
	7.3 -10	0	2	10	3
15-20 kDa	3 - 5.4	0	0	0	2
	5.4 - 7.3	13	15	18	32
	7.3 -10	0	6	5	2
< 15 kDa	3 - 5.4	0	4	0	0
	5.4 - 7.3	8	4	28	13
	7.3 -10	0	5	7	4
Tot. N°		**68**	**67**	**106**	**98**

Analyses were carried out with Image Master software (Amersham Pharmacia Biotech, Uppsala, Sweden).
Spot designation corresponds to those of the gels in Figure 1A-D. Data are the means of three independent experiments ± standard deviations (n = 3).
